# Prognosis of high‐risk human papillomavirus‐related cervical lesions: A hidden Markov model analysis of a single‐center cohort in Japan

**DOI:** 10.1002/cam4.4470

**Published:** 2021-12-17

**Authors:** Ryo Ikesu, Ayumi Taguchi, Konan Hara, Kei Kawana, Tetsushi Tsuruga, Jun Tomio, Yutaka Osuga

**Affiliations:** ^1^ Department of Public Health Graduate School of Medicine The University of Tokyo Bunkyo‐ku Tokyo Japan; ^2^ Department of Obstetrics and Gynecology Graduate School of Medicine The University of Tokyo Bunkyo‐ku Tokyo Japan; ^3^ Department of Economics University of Arizona Tucson Arizona USA; ^4^ Hematology Division Tokyo Metropolitan Cancer and Infectious Diseases Center Komagome Hospital Bunkyo‐ku Tokyo Japan; ^5^ Department of Obstetrics and Gynecology School of Medicine Nihon University Itabashi‐ku Tokyo Japan

**Keywords:** cervical cancer, cervical intraepithelial neoplasia (CIN), hidden Markov model, human papillomavirus (HPV)

## Abstract

**Introduction:**

Previous studies have shown that individuals with human papillomavirus (HPV)‐related cervical lesions have different prognoses according to the HPV genotype. However, these studies failed to account for possible diagnostic misclassification. In this retrospective cohort study, we aimed to clarify the natural course of cervical lesions according to HPV genotype to account for any diagnostic misclassification.

**Materials and Methods:**

Our cohort included 729 patients classified as having cervical intraepithelial neoplasia (CIN). HPV was genotyped in all patients, who were followed up or treated for cervical lesions at the University of Tokyo Hospital from October 1, 2008 to March 31, 2015. Hidden Markov models were applied to estimate the diagnostic misclassification probabilities of the current diagnostic practice (histology and cytology) and the transitions between true states. We then simulated two‐year transition probabilities between true cervical states according to HPV genotype.

**Results:**

Compared with lesions in patients with other HPV genotypes, lesions in HPV 16‐positive patients were estimated to be more likely to increase in severity (i.e., CIN3/cancer); over 2 years, 17.7% (95% confidence interval [CI], 9.3%–29.3%) and 27.8% (95% CI, 16.6%–43.5%) of those with HPV 16 progressed to CIN3/cancer from the true states of CIN1 and CIN2, respectively, whereas 55%–70% of CIN1/2 patients infected with HPV 52/58 remained in the CIN1/2 category. Misclassification was estimated to occur at a rate of 3%–38% in the current diagnostic practice.

**Conclusion:**

This study contributes robust evidence to current literature on cervical lesion prognosis according to HPV genotype and quantifies the diagnostic misclassification of true cervical lesions.

## Graphical abstract

In this retrospective cohort study, hidden Markov models were applied to clarify the natural course of cervical lesions according to human papillomavirus (HPV) genotype to account for any diagnostic misclassification. We revealed: (i) the current diagnostic practice (i.e., histology and cytology) was subject to diagnostic misclassification, and (ii) HPV 16‐positive patients were more likely to progress to more severe lesions than those with other HPV genotypes.

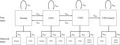

## 1 INTRODUCTION

Cervical cancer is the fourth most common cancer in women worldwide, as approximately 570,000 women developed cervical cancer and 311,000 died of the disease in 2018.^1^ Human papillomavirus (HPV) infection is a common cause of cervical cancer and its precancerous lesions as well as cervical intraepithelial neoplasia (CIN). Previous studies have indicated that cervical lesions should be managed according to HPV genotype.^2^ Besides HPV genotype, patients with cervical lesions are also managed according to their CIN classification (CIN1, CIN2, and CIN3). The risk stratification of patients with cervical lesions according to HPV genotype and CIN classification is critical to present effective treatment strategies while avoiding obstetric complications.^3^, ^4^

In this context, the prognosis of CIN lesions according to HPV genotype has been assessed.^5^ A continuous‐time multistate Markov model was also applied to accommodate the bidirectional feature of CIN lesions^6^; for example, CIN2 can regress to CIN1 or to a normal condition, remain as CIN2, or progress to CIN3 or cervical cancer.^7^ However, another concern when building structural models of CIN lesion prognosis is diagnostic misclassification. Although CIN diagnosis is based on the combination of cytological and histological examinations aided by colposcopy, the accuracy of CIN diagnosis is limited, resulting in the misclassification of the “true” pathology of the lesion. For example, cytologic diagnosis was shown to have low sensitivity in detecting CIN2 or more severe lesions.^8^ Colposcopy‐directed biopsy also tends to underestimate the CIN severity compared with a diagnosis confirmed by surgery.^9^ Nevertheless, few studies have evaluated HPV pathogenesis, accounting for the probability of diagnostic misclassification.^10^

To accommodate these types of measurement challenges, various latent variable models (e.g., factor models and structural equation models) have been adopted in medical research.^11^ Latent variable models can manage unobserved random variables. Recently, another latent variable model, a hidden Markov model, was applied to model (i) the transition between the (unobserved) “true” states and (ii) the probabilities of the “observed” state conditional on the “true” states (misclassification probabilities).^12^, ^13^ In contrast, a Markov model can model only transitions between observed states. In addition to other latent variable models, hidden Markov models have been applied in clinical settings with measurement challenges, such as frailty, HIV infection, and diabetic retinopathy.^14^, ^15^, ^16^

In this study, we applied a hidden Markov model to our cohort of HPV‐infected patients to clarify the natural course of CIN according to HPV genotype, which accounted for the misclassification probability. We aimed to confirm the robustness of the current literature, including a previous study that used a Markov model, on the CIN characteristics according to HPV genotype. Using this model, we also quantified the misclassification probability in CIN diagnosis.

## 2 MATERIALS AND METHODS

### 2.1 Study cohort

Between October 1, 2008 and March 31, 2015, 1427 female patients underwent HPV genotyping at the Obstetrics and Gynecology Department of the University of Tokyo Hospital (Tokyo, Japan). Patients with mild cervical lesions were also followed up at this hospital instead of being referred to other clinics. We reviewed the electronic medical records (EMRs) of those patients and extracted information on pathological (cytological and histological) examinations and treatment of cervical lesions as previously described.^6^

**FIGURE 1:**
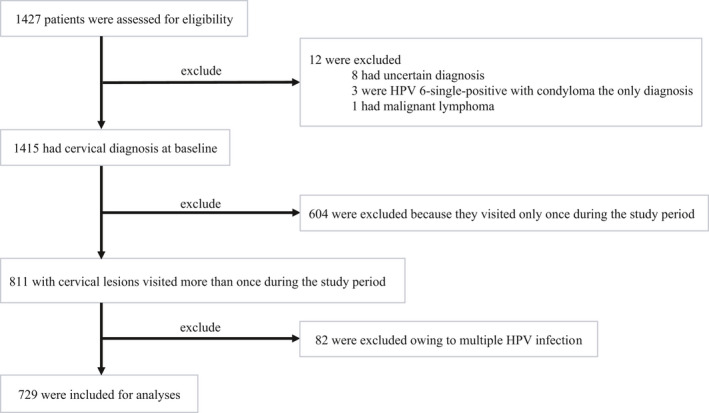
Flowchart illustrating the sample selection

To clarify the natural course of cervical lesions according to the CIN classification, we constructed a dataset that included patients (i) diagnosed with normal cervical lesions, CIN1, or CIN2 at the time of entry and (ii) who visited the Obstetrics and Gynecology Department at least twice during the follow‐up period. Patients were excluded if they had HPV 6‐single‐positive lesions with the sole diagnosis of condyloma during the follow‐up period and if they had only glandular lesions. Patients with multiple HPV genotypes were excluded. One patient with malignant lymphoma was also excluded. Patients were followed up until they received treatment, until they were diagnosed with CIN3 or cervical cancer, until they were moved to another hospital, or until March 31, 2018, whichever occurred first. Finally, 729 patients (6082 observations) were included in the dataset (Figure 1).

This study was performed under the principles of the Declaration of Helsinki and was approved by the ethics committee of the Graduate School of Medicine, University of Tokyo (nos. 1390–1, G10082‐11, and G0637‐8).

### 2.2 Variables

#### 2.2.1 HPV genotype

A cervical swab sample was collected from each patient at the time of entry or at the first visit when the HPV DNA genotype was determined. DNA was extracted from the cervical samples with a DNeasy Blood Mini Kit (Qiagen), the procedure for which has been previously described.^6^ For each patient, we confirmed the HPV genotype, which was recorded in the EMRs. Since the genotyping was performed only once for each patient, the HPV genotype did not change over time. We classified HPVs into Group 1 or Group 2A (HPVs 16, 18, 31, 33, 35, 39, 45, 51, 52, 56, 58, 59, and 68) as “high‐risk HPVs” (hrHPVs) according to the classification of the International Agency for Research on Cancer.^17^ We focused on HPVs 16, 18, 52, and 58.^6^ hrHPVs other than these four genotypes were defined as “other hrHPVs.” The remaining HPVs were classified as “no hrHPVs.” Patients without HPV infection were placed into this category (i.e., “no hrHPV” patients).

#### 2.2.2 Pathological diagnosis

The dates of visits and the trajectory of cytological and histological results were maintained in the EMRs of each patient. At the patient’s first visit, we collected a histological sample via colposcopy. Thereafter, we performed repeated cytological and/or histological examinations in line with each patient’s visits. The histological examination was performed at the gynecologists’ discretion, especially when colposcopic findings indicated severe lesions compared with the previous diagnosis, the disease had progressed since the previous assessment, or when lesions that tended to exhibit discrepancies in cytological and histological diagnoses were observed.

Based on the cytological and histological results obtained after each visit, we grouped the pathological diagnoses at each visit into one of the following four CIN categories: normal, CIN1, CIN2, and CIN3/cancer. For some controversial records, the investigators (gynecologic oncology experts) discussed and determined the following: (i) CIN1–2 was classified as CIN1, (ii) CIN2–3 was classified as CIN2, (iii) uncertain diagnoses (e.g., atypical squamous cells of uncertain significance, atypical squamous cells that cannot exclude high‐grade squamous intraepithelial lesion [HSIL], and dysplasia without grading) were excluded from diagnostic reliability, and (iv) if the histological and cytological results were not identical, we accepted the more severe result as the diagnosis. We classified CIN1–2 as CIN1 and CIN2–3 as CIN2 in order to mitigate the overdiagnosis from the fourth protocol.

Furthermore, based on the dates of patients’ visits, we calculated the follow‐up time at the time of each examination. The age of the patients at the time of entry was extracted from the EMRs.

### 2.3 Continuous‐time multistate hidden Markov model

We applied the continuous‐time multistate hidden Markov model to account for the possibility that pathological examinations were subject to misclassification and for the natural bidirectional course of cervical lesions. This statistical model contains two parts: (i) the transition between the “true” pathological states and (ii) the probabilities of the “observed” diagnosis conditional on the “true” pathological states (misclassification probabilities).^13^, ^18^

**FIGURE 2:**
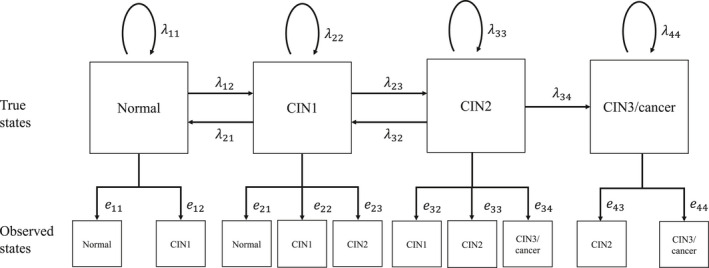
Hidden Markov model for the underlying true pathological states (CIN categorization). We defined four states: normal (state 1), cervical intraepithelial neoplasia 1 (CIN1, state 2), CIN2 (state 3), and CIN3/cancer (state 4). The arrows in this figure specify possible transitions among these states; all transitions between adjacent states were allowed, except for the transition from CIN3/cancer to CIN2 (i.e., CIN3/cancer was the absorbing state). The transition parameter $\lambda_{\mathrm{ij}}$ represents an instantaneous rate of transition from the true pathological state *i* to the true pathological state *j*. $e_{\mathrm{rs}}$ denotes the probability of observing the state *s* conditionally on the true state *r* ($e_{11}$ = 1−$e_{12}$, $e_{22}$ = 1−$e_{21}$−$e_{23}$, $e_{33}$ = 1−$e_{32}\;-\;e_{34}$, $e_{44}$ = 1−$e_{43}$)

We assumed transitions of the “true” pathological states and the misclassification patterns as shown in Figure 2. For each of the underlying true states and the observed states, the state space was {Normal, CIN1, CIN2, and CIN3/cancer} with each element corresponding to state 1, state 2, state 3, and state 4, respectively. Each observed state was determined by the underlying true state and misclassification. All transitions between adjacent true states were allowed in this model, except for the transition from CIN3/cancer to CIN2, as CIN3/cancer was the absorbing state. The transition parameter $\lambda_{\mathrm{ij}}$ represents the transition intensity, which can be interpreted as an instantaneous rate of transition from the true pathological state *i* to the true pathological state *j* (e.g., $\lambda_{23}$ denotes the transition intensity from true CIN1 to true CIN2). Based on the clinical assumption that adjacent misclassifications could account for most diagnostic misclassifications, we assumed the following misclassification matrix for the estimation (Figure 2):

$$E=\left(\begin{array}{cccc} 1‐e_{12} & e_{12} & 0 & 0\\ e_{21} & 1‐e_{21}‐e_{23} & e_{23} & 0\\ 0 & e_{32} & 1‐e_{32}‐e_{34} & e_{34}\\ 0 & 0 & e_{43} & 1‐e_{43} \end{array}\right)$$

where $e_{\mathrm{rs}}$ denotes the probability of observing the state *s* conditionally on the true state *r* (e.g., $e_{34}$ denotes the probability of observing CIN3 when the true state is CIN2). In other words, we allowed for just a “one‐step” misclassification adjacent to the true state.

### 2.4 Statistical analysis

First, we reported the summary statistics of patients according to HPV genotype (HPV 16, 18, 52, 58, other hrHPVs, and no hrHPVs) and observed diagnoses at the time of entry. We also showed the transition of diagnoses according to HPV genotype over two successive examinations.

Next, a maximum likelihood procedure was applied to estimate the parameters $\lambda_{\mathrm{ij}}$ and $e_{\mathrm{rs}}$ using the *msm* package in R.^18^ The estimates were derived to maximize a likelihood function composed of the transition probabilities among the true states and the misclassification probabilities conditional on the true state. Along with the parameter estimation, the “true” initial distribution was also estimated in the models. The detailed formulation of the likelihood function is described elsewhere.^18^ To account for possible differences in the natures of HPV genotypes, we introduced dummy variables representing each HPV genotype (except for “no hrHPVs,” the reference genotype). These dummy variables were included in the model as covariates for the transition parameter $\lambda_{\mathrm{ij}}$. Furthermore, we simulated the probabilities of transitions among the true states for up to 2 years. We selected the span of 2 years for two reasons. First, a two‐year transition prediction is considered a good benchmark for the prognosis of hrHPV‐related cervical lesions. At least half of those lesions have been reported to regress to a normal state and approximately 10% progress to CIN3 within 2 years.^5^ Second, the median follow‐up period of our subjects was approximately three years. Hence, a transition prediction beyond this period was unwarranted. Finally, we evaluated our model by visually comparing the prevalence of observed diagnoses and that of simulated observed diagnoses, which was derived by right‐multiplying the simulated “true” prevalence matrix by the estimated misclassification matrix. R 3.6.2 (R Foundation) was used for all analyses.^19^

### 2.5 Sensitivity analysis

To confirm the robustness of our primary analysis, we performed two different sensitivity analyses. First, we assumed another misclassification matrix for the CIN categorization. Specifically, we set $e_{34}$ in the misclassification matrix to zero. We performed this sensitivity analysis because the estimation of the parameter $e_{43}$ jointly with $e_{34}$ in the CIN analysis was supposedly unstable due to the nature of our dataset. Since the analysis of patients in our dataset with the CIN categorization was truncated once they were diagnosed with CIN3/cancer, the available observations to estimate $e_{43}$ were reasonably scarce. Using this sensitivity analysis, we aimed to stabilize the estimation of $e_{43}$ to examine whether the estimates in the primary analysis were subject to this instability.

Second, to clarify the natural course of cervical lesions according to the two‐tier classification (World Health Organization 2020),^20^ we constructed another dataset based on that classification (i.e., normal, low‐grade squamous intraepithelial lesion [LSIL], and HSIL). The details of this dataset are described in the Supplemental material (eAppendix 1, Figure S1). We applied the same analysis as in the CIN categorization for the two‐tier classification according to the model specified in Figure S2. For these sensitivity analyses, the estimated misclassification matrix was presented. The probabilities of transitions among the true states up to two years were then simulated.

## 3 RESULTS

### 3.1 Patient characteristics

Table 1 shows the characteristics of our study subjects. In total, 729 patients in the CIN category were enrolled with a mean follow‐up of 3.3 years (standard deviation [SD]: 2.4 years). The mean age at the time of entry was 39.1 years (SD: 9.8 years), and the mean number of visits was 8.3 (SD: 5.4). At the time of entry, 185 (25.3%), 270 (37.0%), and 274 (37.6%) patients were diagnosed as normal or with CIN1 or CIN2, respectively.

**TABLE 1:** Basic characteristics of the study subjects at the time of entry (CIN categorization)

Diagnosis at the time of entry		HPV 16	HPV 18	HPV 52	HPV 58	Other hrHPVs	No hrHPVs	All
Normal	N	8	7	14	10	24	122	185
	Age at entry (years), mean (SD)	39.9 (8.2)	45.3 (15.9)	38.0 (10.1)	44.9 (17.4)	42.7 (16.3)	41.2 (10.5)	41.5 (11.9)
	Number of visits, mean (SD)	6.6 (5.8)	8.4 (2.7)	8.9 (7.2)	7.0 (5.0)	8.0 (4.8)	6.5 (4.0)	7.0 (4.5)
	Follow‐up interval (years), mean (SD)	0.47 (0.35)	0.48 (0.30)	0.51 (0.37)	0.49 (0.44)	0.47 (0.27)	0.52 (0.39)	0.50 (0.36)
	Follow‐up period (years), mean (SD)	2.7 (3.0)	3.4 (1.1)	4.3 (3.3)	3.0 (2.4)	3.4 (2.2)	3.1 (2.1)	3.2 (2.3)
CIN1	N	20	8	32	23	67	120	270
	Age at entry (years), mean (SD)	35.3 (9.2)	33.0 (10.6)	39.1 (8.7)	36.7 (8.3)	34.5 (7.1)	38.8 (10.1)	37.1 (9.2)
	Number of visits, mean (SD)	8.8 (5.4)	6.5 (2.8)	9.4 (5.0)	11.0 (5.5)	9.4 (4.4)	9.3 (5.4)	9.3 (5.1)
	Follow‐up interval (years), mean (SD)	0.38 (0.19)	0.51 (0.56)	0.38 (0.21)	0.42 (0.28)	0.38 (0.18)	0.42 (0.30)	0.41 (0.27)
	Follow‐up period (years), mean (SD)	3.5 (2.6)	3.0 (1.8)	3.6 (2.1)	4.2 (2.5)	3.6 (2.0)	3.9 (2.4)	3.7 (2.3)
CIN2	N	64	12	52	37	51	58	274
	Age at entry (years), mean (SD)	38.4 (7.8)	42.5 (5.7)	41.4 (8.0)	41.1 (8.1)	39.1 (7.8)	37.0 (9.3)	39.4 (8.3)
	Number of visits, mean (SD)	6.4 (5.9)	6.7 (5.2)	8.5 (6.3)	8.4 (5.6)	8.7 (5.7)	9.4 (5.7)	8.1 (5.9)
	Follow‐up interval (years), mean (SD)	0.33 (0.19)	0.31 (0.11)	0.35 (0.19)	0.36 (0.22)	0.38 (0.41)	0.37 (0.35)	0.35 (0.28)
	Follow‐up period (years), mean (SD)	1.9 (2.3)	1.9 (1.9)	2.9 (2.5)	3.1 (2.4)	3.2 (2.2)	3.5 (2.4)	2.8 (2.4)

Note

Other hrHPVs included HPV 31, 33, 35, 39, 45, 51, 56, 59, and 68. No hrHPVs were HPVs other than HPV 16, 18, 52, 58, or other hrHPVs.

Abbreviations: hrHPV, high‐risk human papillomavirus; SD, standard deviation.

Table 2 shows the visit‐wise transitions of observed lesions according to HPV genotype. For the CIN categorization, 5353 transitions were observed, of which 551, 165, 770, 569, 1130, and 2168 transitions were observed for HPVs 16, 18, 52, 58, other hrHPVs, and no hrHPVs, respectively. The HPV genotype pattern in transitions was similar for the two‐tier classification. Most (78%–90%) patients in the normal state remained in that state over consecutive observations (e.g., 86.7% of HPV 16‐positive patients in the normal state observed at a certain visit were also categorized as normal at the next visit). The progression from CIN2 to CIN3 was dependent on HPV genotype: 11.9%, 7.5%, 5.1%, 3.7%, 5.4%, and 2.0% of those with HPVs 16, 18, 52, 58, other hrHPVs, and no hrHPVs, progressed to CIN3, respectively.

**TABLE 2:** Transitions from each diagnosis of cervical epithelial lesions according to HPV genotype (CIN categorization)

Diagnosis at (t − 1) visit	HPV category	Diagnosis at t visit				
		Normal	CIN1	CIN2	CIN3	Cancer
Normal	HPV 16	190 (86.7)	10 (4.5)	16 (7.3)	3 (1.3)	0 (0.0)
	HPV 18	60 (80.0)	11 (14.6)	4 (5.3)	0 (0.0)	0 (0.0)
	HPV 52	251 (77.7)	44 (13.6)	26 (8.0)	2 (0.6)	0 (0.0)
	HPV 58	196 (84.1)	21 (9.0)	14 (6.0)	2 (0.8)	0 (0.0)
	Other hrHPVs	573 (86.8)	71 (10.7)	14 (2.1)	2 (0.3)	0 (0.0)
	No hrHPVs	1348 (89.9)	119 (7.9)	28 (1.8)	3 (0.2)	1 (0.0)
CIN1	HPV 16	24 (26.9)	30 (33.7)	33 (37.0)	2 (2.2)	0 (0.0)
	HPV 18	15 (40.5)	13 (35.1)	8 (21.6)	1 (2.7)	0 (0.0)
	HPV 52	65 (33.6)	80 (41.4)	46 (23.8)	2 (1.0)	0 (0.0)
	HPV 58	39 (26.5)	69 (46.9)	36 (24.4)	3 (2.0)	0 (0.0)
	Other hrHPVs	122 (45.3)	113 (42.0)	33 (12.2)	1 (0.3)	0 (0.0)
	No hrHPVs	225 (52.5)	162 (37.8)	38 (8.8)	3 (0.7)	0 (0.0)
CIN2	HPV 16	26 (10.6)	34 (13.9)	153 (62.9)	29 (11.9)	1 (0.4)
	HPV 18	7 (13.2)	6 (11.3)	36 (67.9)	4 (7.5)	0 (0.0)
	HPV 52	36 (14.1)	44 (17.3)	161 (63.3)	13 (5.1)	0 (0.0)
	HPV 58	21 (11.1)	43 (22.7)	118 (62.4)	7 (3.7)	0 (0.0)
	Other hrHPVs	40 (19.9)	35 (17.4)	115 (57.2)	11 (5.4)	0 (0.0)
	No hrHPVs	57 (23.6)	42 (17.4)	137 (56.8)	5 (2.0)	0 (0.0)

Note

Values are the number (percentage) of observed transitions from a visit to the next visit.

Other hrHPVs included HPV 31, 33, 35, 39, 45, 51, 56, 59, and 68.

No hrHPVs were HPVs other than HPV 16, 18, 52, 58, or other hrHPVs.

Abbreviations: hrHPV: high‐risk human papillomavirus.

### 3.2 Primary analysis

Table 3 represents our estimates for the misclassification matrix for the CIN categorization. Of patients with the normal state as their true state, 95.7% (95% confidence interval [CI], 94.2%–96.9%) were estimated to be diagnosed correctly. The model estimated that, of patients with the true state of CIN1, 61.9% (95% CI, 51.6%–71.2%) were diagnosed correctly, while 24.4% (95% CI, 17.3%–33.4%) and 13.5% (95% CI, 9.3%–19.2%) were misclassified as having normal and CIN2, respectively. For those with the true state of CIN2, 88.7% (95% CI, 80.4%–93.8%) were estimated to be diagnosed correctly as having CIN2, while 6.2% (95% CI, 3.8%–10.0%) and 4.9% (95% CI, 3.1%–7.7%) were estimated to be misclassified as having CIN1 and CIN3/cancer, respectively. For those with the true state of CIN3/cancer, 95.5% (95% CI, 24.6%–99.9%) were estimated to be diagnosed correctly. However, this wide 95% CI implied the instability of the estimation.

**TABLE 3:** Misclassification probabilities for the CIN categorization based on cytology and histology

True underlying state	Observed state			
	Normal	CIN1	CIN2	CIN3/cancer
Normal	0.957 (0.942–0.969)	0.042 (0.030–0.057)	0.000 (0.000–0.000)	0.000 (0.000–0.000)
CIN1	0.244 (0.173–0.334)	0.619 (0.516–0.712)	0.135 (0.093–0.192)	0.000 (0.000–0.000)
CIN2	0.000 (0.000–0.000)	0.062 (0.038–0.100)	0.887 (0.804–0.938)	0.049 (0.031–0.077)
CIN3/cancer	0.000 (0.000–0.000)	0.000 (0.000–0.000)	0.044 (0.000–0.753)	0.955 (0.246–0.999)

Note

Values are the estimated emission probabilities (95% confidence interval).

Table 4 shows the predicted transition probabilities of true lesions after two years according to HPV genotype. For HPV 16, 2.3% (95% CI, 0.9%–5.5%), 17.7% (95% CI, 9.3%–29.3%), and 27.8% (95% CI, 16.6%–43.5%) of patients progressed to CIN3/cancer from the true state of normal, CIN1, and CIN2, respectively. On the contrary, 44.4% (95% CI, 32.5%–55.5%) and 29.9% (95% CI, 20.3%–39.9%) of HPV 16‐positive patients regressed to a normal state from CIN1 and CIN2, respectively. For HPV 18‐positive patients, the progression to CIN3/cancer was less likely than for HPV 16‐positive patients; 0.7% (95% CI, 0.0%–14.4%) transitioned from a normal state, 2.6% (95% CI, 0.0%–35.2%) transitioned from CIN1, and 10.9% (95% CI, 0.0%–96.5%) transitioned from CIN2. For HPV 52/58‐positive patients, the transitions were more likely to be stable over the 2 years than for HPV 16/18‐positive patients; approximately 55%–70% of CIN1/2 patients remained as CIN1/2 after 2 years. Figure S3 illustrates the observed prevalence of the test‐revealed lesions and the simulated prevalence of observed lesions by HPV genotype.

**TABLE 4:** Predicted two‐year transition probabilities according to HPV genotype (CIN categorization)

Current state	HPV category	State after two years			
		Normal	CIN1	CIN2	CIN3/cancer
Normal	HPV 16	0.832 (0.685–0.913)	0.064 (0.033–0.126)	0.079 (0.036–0.155)	0.023 (0.009–0.055)
	HPV 18	0.736 (0.493–0.897)	0.159 (0.054–0.310)	0.096 (0.006–0.214)	0.007 (0.000–0.144)
	HPV 52	0.933 (0.168–0.998)	0.045 (0.001–0.475)	0.020 (0.000–0.312)	0.001 (0.000–0.037)
	HPV 58	0.974 (0.050–0.999)	0.017 (0.000–0.581)	0.007 (0.000–0.324)	0.000 (0.000–0.052)
	Other hrHPVs	0.889 (0.808–0.940)	0.090 (0.049–0.157)	0.017 (0.008–0.036)	0.002 (0.000–0.007)
	No hrHPVs	0.910 (0.768–0.966)	0.068 (0.025–0.172)	0.019 (0.006–0.050)	0.001 (0.000–0.005)
CIN1	HPV 16	0.444 (0.325–0.555)	0.127 (0.085–0.194)	0.250 (0.168–0.339)	0.177 (0.093–0.293)
	HPV 18	0.622 (0.385–0.752)	0.161 (0.075–0.314)	0.189 (0.007–0.370)	0.026 (0.000–0.352)
	HPV 52	0.392 (0.153–0.485)	0.317 (0.246–0.479)	0.256 (0.186–0.356)	0.033 (0.010–0.105)
	HPV 58	0.410 (0.050–0.508)	0.330 (0.241–0.569)	0.238 (0.145–0.365)	0.020 (0.002–0.175)
	Other hrHPVs	0.734 (0.648–0.791)	0.171 (0.124–0.232)	0.071 (0.038–0.111)	0.023 (0.009–0.055)
	No hrHPVs	0.673 (0.614–0.712)	0.189 (0.159–0.239)	0.117 (0.091–0.145)	0.020 (0.009–0.041)
CIN2	HPV 16	0.299 (0.203–0.399)	0.137 (0.091–0.200)	0.285 (0.189–0.389)	0.278 (0.166–0.435)
	HPV 18	0.285 (0.024–0.484)	0.143 (0.006–0.267)	0.461 (0.000–0.730)	0.109 (0.000–0.965)
	HPV 52	0.236 (0.103–0.317)	0.349 (0.265–0.461)	0.334 (0.229–0.438)	0.079 (0.026–0.240)
	HPV 58	0.252 (0.041–0.345)	0.372 (0.242–0.514)	0.323 (0.150–0.443)	0.052 (0.006–0.407)
	Other hrHPVs	0.437 (0.316–0.546)	0.219 (0.155–0.271)	0.195 (0.099–0.313)	0.146 (0.065–0.308)
	No hrHPVs	0.406 (0.334–0.468)	0.250 (0.213–0.290)	0.253 (0.189–0.326)	0.089 (0.044–0.179)

Note

Values are the predicted transition probabilities (95% confidence interval) from the current true lesions over two years.

Other hrHPVs included HPV 31, 33, 35, 39, 45, 51, 56, 59, and 68.

No hrHPVs were HPVs other than HPV 16, 18, 52, 58, or other hrHPVs.

Abbreviation: hrHPV, high‐risk human papillomavirus.

### 3.3 Sensitivity analysis

Tables S1 and S2 show the misclassification matrix and predicted transition probabilities of true lesions for the sensitivity analysis, respectively. The results of this sensitivity analysis were consistent with those of our primary analysis for CIN categorization. Furthermore, compared with our primary analysis, the sensitivity analysis resulted in narrower confidence intervals for the misclassification matrix (Table 3 and Table S1).

For the two‐tier classification, dataset characteristics and visit‐wise transitions of observed lesions are shown in Tables S3 and S4, respectively. Table S5 contains our estimates for the misclassification matrix in the two‐tier classification. Of patients with a normal state as their true state, 96.6% (95% CI, 95.1%–97.7%) were estimated to be diagnosed correctly. It was estimated that for those with the true state of LSIL, 61.6% (95% CI, 52.8%–69.8%) were diagnosed correctly, while 24.7% (95% CI, 19.3%–31.0%) and 13.5% (95% CI, 9.8%–18.4%) were misclassified as normal and having HSIL, respectively. For those with the true state of HSIL, 95.4% (95% CI, 93.0%–97.1%) were estimated to be diagnosed correctly.

Table S6 shows the predicted transition probabilities of true lesions after two years according to HPV genotype. For HPV 16, 10.7% (95% CI, 5.7%–19.6%) and 41.6% (95% CI, 31.3%–52.0%) of patients progressed to HSIL from the true states of normal and LSIL, respectively. In contrast, 44.8% (95% CI, 33.8%–55.5%) and 22.4% (95% CI, 15.1%–30.5%) of HPV 16‐positive patients regressed to a normal state from LSIL and HSIL, respectively. The progression to HSIL was less likely in HPV 18‐positive patients compared with HPV 16‐positive patients; 4.6% (95% CI, 0.7%–17.8%) from a normal state and 20.3% (95% CI, 7.3%–40.7%) from LSIL. The transitions were more likely to be stable over two years in HPV 52/58‐positive patients than in HPV 16/18‐positive patients; approximately 30%–35% of LSIL patients remained in the same state after 2 years. Figure S4 illustrates the observed prevalence of the test‐revealed lesions and the simulated prevalence of observed lesions by HPV genotype.

## 4 DISCUSSION

In this study, we applied the continuous‐time multistate hidden Markov model and successfully estimated the transition probabilities of cervical lesions according to HPV genotype, which accommodated the misclassification probabilities of pathological lesions. Adopting a latent variable approach (i.e., hidden Markov model) as in previous medical research, we revealed the following two points: (i) the current diagnostic practice (i.e., histology and cytology) was subject to diagnostic misclassification at a rate of 3%–38% and (ii) even when the diagnostic misclassification was accounted for, HPV 16‐positive patients were more likely to progress to more severe lesions (i.e., CIN3/cancer or HSIL) than those with other HPV genotypes, which was consistent with previous studies.^5^, ^6^

Our results showed that even when we accounted for the diagnostic misclassification in current practice, the progression rate toward CIN3/cancer was higher among HPV 16‐ positive patients than among others. In contrast, patients with HPV 52/58 tended to remain in the CIN1/2 category. Moreover, the progression and regression rates were similar between patients with other hrHPVs and those with no hrHPVs. These results are consistent with those seen in the literature and corroborate the finding that patients with HPV 16 are more likely to develop more severe lesions than those with HPV 52, HPV 58, other hrHPVs, or no hrHPVs.^6^ By applying the hidden Markov model to accommodate the diagnostic misclassification, our study confirmed the robustness of the current literature on CIN characteristics according to HPV genotype. Our results were robust regardless of the cervical lesion categorization. We performed a sensitivity analysis for another dataset based on the two‐tier classification and the results were similar to those of our primary analysis as follows: HPV 16‐positive patients were more likely to progress to severer lesions (i.e., HSIL) than those with other HPV genotypes.

Our prognostic prediction was different from that in a previous study, which used a continuous‐time multistate Markov model.^6^ Notably, patients with HPV 52/58‐derived CIN1/2 were more likely to be classified as having CIN1 over two years than in the previous study. Furthermore, compared with the corresponding women in the previous study, those in the initial normal state in this study were more likely to remain in the normal state over two years (e.g., 83.2% vs. 59.8% for HPV 16). This discrepancy could be attributed to the compositional difference of the initial states between the two studies, as the diagnostic misclassification was accounted for in this study; for example, the normal state in the previous study was reasonably composed of patients with true normal and more severe states. This accommodation of diagnostic misclassification also helped us reveal the stable nature of CIN1/2 in HPV 52/58‐positive patients. Although the slightly different CIN definitions between the studies and the (non‐)existence of an absorbing state in those models might explain the different results, our model, which incorporates diagnostic misclassification, could successfully derive a better picture of cervical lesion prognosis according to CIN classification and HPV genotype.

Our results estimated that diagnosis through histological and cytological examinations was subject to misclassification at a rate of 3%–38%. These misclassifications can be explained by two mechanisms. First, histological and cytological examinations may be subject to sampling errors. The diagnostic precision of histological examination has been shown to depend on the quality of specimen processing.^21^, ^22^, ^23^ Furthermore, it is sometimes difficult to distinguish metaplastic epithelial cells from atypical epithelial cells in a cytological examination.^24^, ^25^, ^26^ Second, cervical lesions are representative of a continuous disease spectrum rather than discrete states. Categorizing this continuous spectrum into discrete systems (i.e., CIN classification and the two‐tier classification) can underestimate the extent of variation in the disease spectrum (i.e., loss of information), which may result in diagnostic misclassification.^27^, ^28^

To the best of our knowledge, this is the first study to apply a hidden Markov model to quantify the misclassification probabilities of the cervical diagnostic procedure as well as the natural history of HPV infections; Kang and Lagakos applied a hidden Markov model only to the natural history of HPV infections under the prespecified misclassification probability.^10^ Notably, the misclassification probability in patients with the true state of CIN1 was higher than those with the other true states (37.9% for CIN1 vs. 4.2%–11.1% for the other true states). This result was biologically plausible, as CIN1 is a low‐grade lesion with a high propensity for regression due to immune responses, but this lesion type may also progress to a high‐grade lesion.^29^, ^30^, ^31^ The high misclassification probabilities in CIN1 patients may reflect the biological instability of these lesions due to the smooth transition imposed by the hidden Markov model. In contrast, the misclassification probability of observing CIN2 in patients with the true state of CIN3/cancer was only 4.4% (11% for the sensitivity analysis). To analyze the clinical implications, we applied Bayes’ theorem to the misclassification estimate of observing CIN2 with CIN3/cancer as the true state; we then calculated the probability of the true state of CIN3/cancer when CIN2 was observed. We may assume subjectively that the marginal probabilities of observing CIN2 and the true state of CIN3/cancer are 40% and 10%, respectively. Then, Bayes’ theorem indicates that the probability of the true state of CIN3/cancer is 1.1% (0.044*0.1/0.4 = 0.011 or 1.1%; 2.8% for the sensitivity analysis). Although the “true state” estimated from the hidden Markov model was not the “clinical true state” that was histologically validated by conization or total hysterectomy, this small probability of overlooking true CIN3/cancer with CIN2 observed may justify the current Japanese practice, where patients with (observed) CIN2 are carefully followed up without treatment. To further validate this practice, an attempt to predict a patient's true state more accurately may be a valid direction for future work.

Some limitations of this study should be acknowledged. First, we could not accommodate the effect of concurrent multiple HPV infections on the progression and regression of cervical lesions. Even for interaction terms between two different HPV genotypes, our cohort was too small to account for multiple HPV infections. Compared with single infections, coinfections with multiple α9 species have been shown to be associated with an increased risk of CIN2 or more severe lesions.^32^ Second, because the genotyping was performed only once at the baseline and the sample size was not large enough, we could not investigate the temporary transitions of HPV infections, which could affect the progression and regression of cervical lesions.^33^, ^34^ Third, we could not account for patient heterogeneity in terms of various factors, such as age, sexual activity, menopausal status, contraception, and HPV vaccination. It is possible that these patients’ characteristics can affect the pathogenesis of cervical lesions or the precision of the diagnosis.^35^, ^36^, ^37^, ^38^, ^39^, ^40^, ^41^ However, we did not have information on sexual activity, menopausal status, contraception, or HPV vaccination. Preliminary analyses in which we included age as a covariate was unsuccessful in estimation convergence. We could not include the heterogeneity by HPV genotypes for estimating the initial distribution of cervical lesions (i.e., normal, CIN1, and CIN2) while achieving the model convergence. Nevertheless, the impacts of some of these characteristics should be minor in this study for the following reasons: (i) oral contraception was not prevalent in Japan during the study period^42^; (ii) HPV vaccination coverage was considered to be low in our cohort, because free vaccination against HPV began in 2010, particularly aiming for girls aged 12–16 years, and very few patients in our cohort (median age at the time of entry: 37.9 years [interquartile range: 32.1–44.7 years]) were eligible for it.^43^ Further studies with a larger number of participants are expected to accommodate these covariates in a hidden Markov model.

Fourth, our model specification might not be optimal. Our estimation in the analysis of the CIN categorization was unstable due to the nature of our dataset. Specifically, the confidence intervals were wide for the misclassification probability with the true underlying state CIN3/cancer, since the analysis of patients in our dataset with the CIN categorization was truncated once they were diagnosed with CIN3/cancer; this dataset structure increased the difficulty in estimating both the probability of observing CIN2 in patients believed to have CIN3/cancer (i.e., $e_{43}$) and the probability of observing CIN3/cancer in patients with CIN2 (i.e., $e_{34}$) in the same model. Furthermore, only “one‐step” misclassifications were allowed in this study, although other misclassifications were possible. Misclassification heterogeneity may have been present due to different diagnostic procedures (i.e., cytologic and histologic examinations) or physicians in charge of patients. In our preliminary analyses, most of the more generous misclassification patterns did not achieve estimation convergence. Despite these concerns, our analyses should provide reliable estimates: (i) we confirmed that our results were robust to the alternative specification of the misclassification matrix, where the confidence intervals were much smaller, and (ii) our model specification with just “one‐step” misclassifications is an appropriate abstraction of the diagnostic reality if other misclassifications were sufficiently rarer than these misclassifications.

Fifth, our model assumed that the transition intensities were independent of the time spent in each state. Previous studies have proposed time‐inhomogeneous transition model^10^, ^44^; however, our cohort was too small to implement it. Future studies are expected to explore the possible time‐inhomogeneous transition intensities between cervical lesions.

Lastly, this was a single‐institution study. Patients included in this study were restricted to those who previously had abnormal cytology and who had visited the university hospital. Hence, the generalizability of our results to other settings warrants further research.

In conclusion, we applied a continuous‐time multistate hidden Markov model to reveal the different prognosis of cervical lesions according to HPV genotype; HPV 16‐positive patients were more likely to progress to CIN3/cancer than those with other HPV genotypes, and those with HPV 52/58‐derived CIN1/2 tended to remain in the CIN1 category. We believe this study contributes robust evidence to the current literature on cervical lesion prognosis according to HPV genotype and quantifies the diagnostic misclassification of true cervical lesions.

## CONFLICT OF INTEREST

The authors declare no conflict of interest.

## AUTHOR CONTRIBUTIONS

Conceptualization, Ryo Ikesu, Ayumi Taguchi, Konan Hara, and Kei Kawana; methodology, Ryo Ikesu, Ayumi Taguchi, and Konan Hara; data collection and data curation, Ayumi Taguchi and Tetsushi Tsuruga; supervision, Kei Kawana, Tetsushi Tsuruga, Jun Tomio, and Yutaka Osuga; funding acquisition, Ayumi Taguchi. All authors reviewed the manuscript and edited it for intellectual content and gave final approval for this version.

## ETHICAL APPROVAL STATEMENT

The Ethics Committee of the Graduate School of Medicine, University of Tokyo approved this study (nos. 1390–1, G10082‐11, and G0637‐8), which was performed in accordance with the Declaration of Helsinki.

## CONSENT FOR PUBLICATION

All the patients included in this study provided consent for the publication of the research findings.

## Supporting information

Fig S1

Fig S2

Fig S3

Fig S4

Table S1‐S6

Appendix

## Data Availability

The data supporting the findings of this study are available upon reasonable request.
