# Supplementary figures and images for: Prognosis of high‐risk human papillomavirus‐related cervical lesions: A hidden Markov model analysis of a single‐center cohort in Japan
Source: Cancer Med. 2021 Dec 17;11(3):664–75. doi: 10.1002/cam4.4470 (PMC8817087; doi:10.1002/cam4.4470)

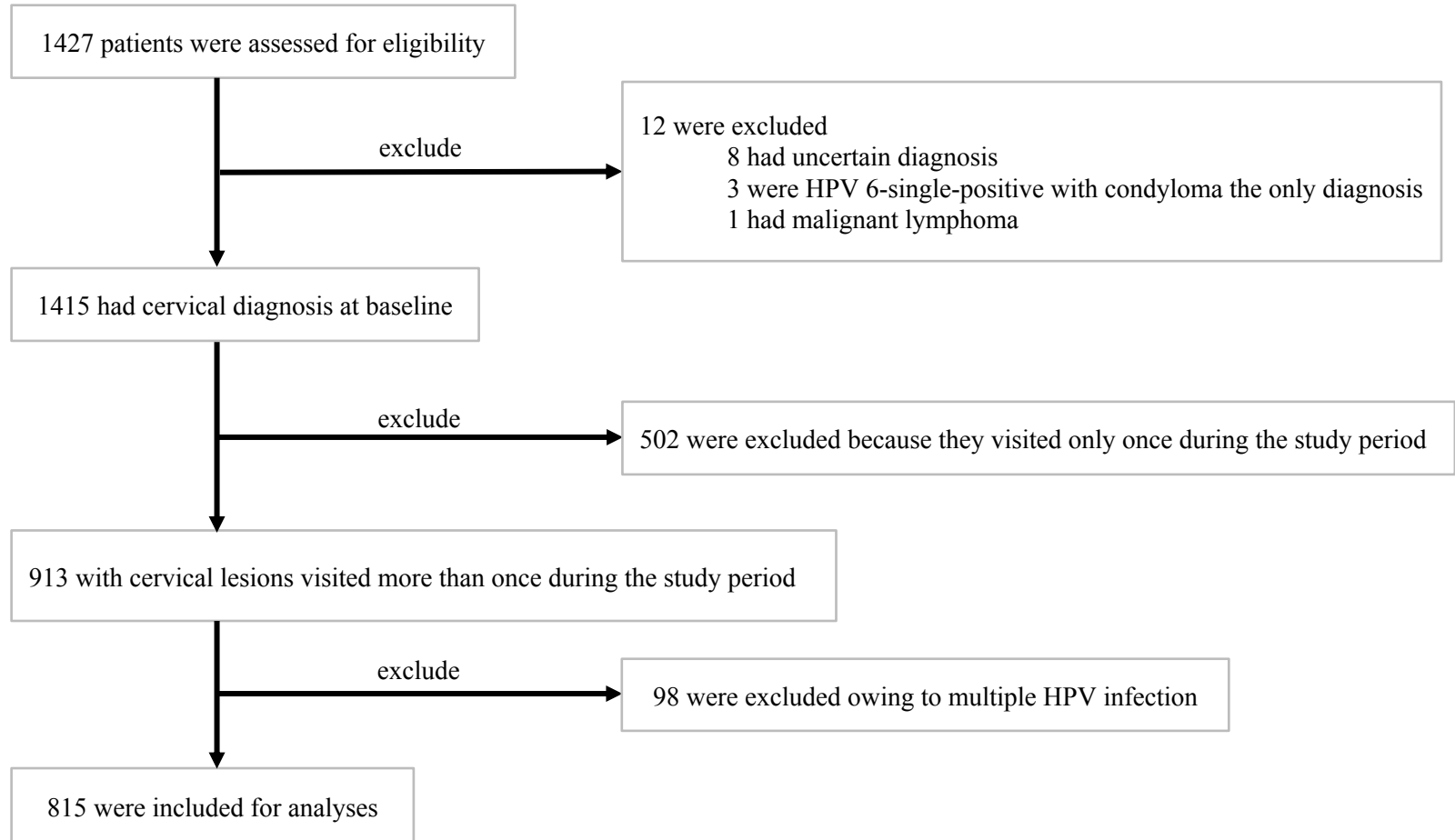

Supplement: Supplementary file 1 — Fig S1 [file CAM4-11-664-s005.pdf]

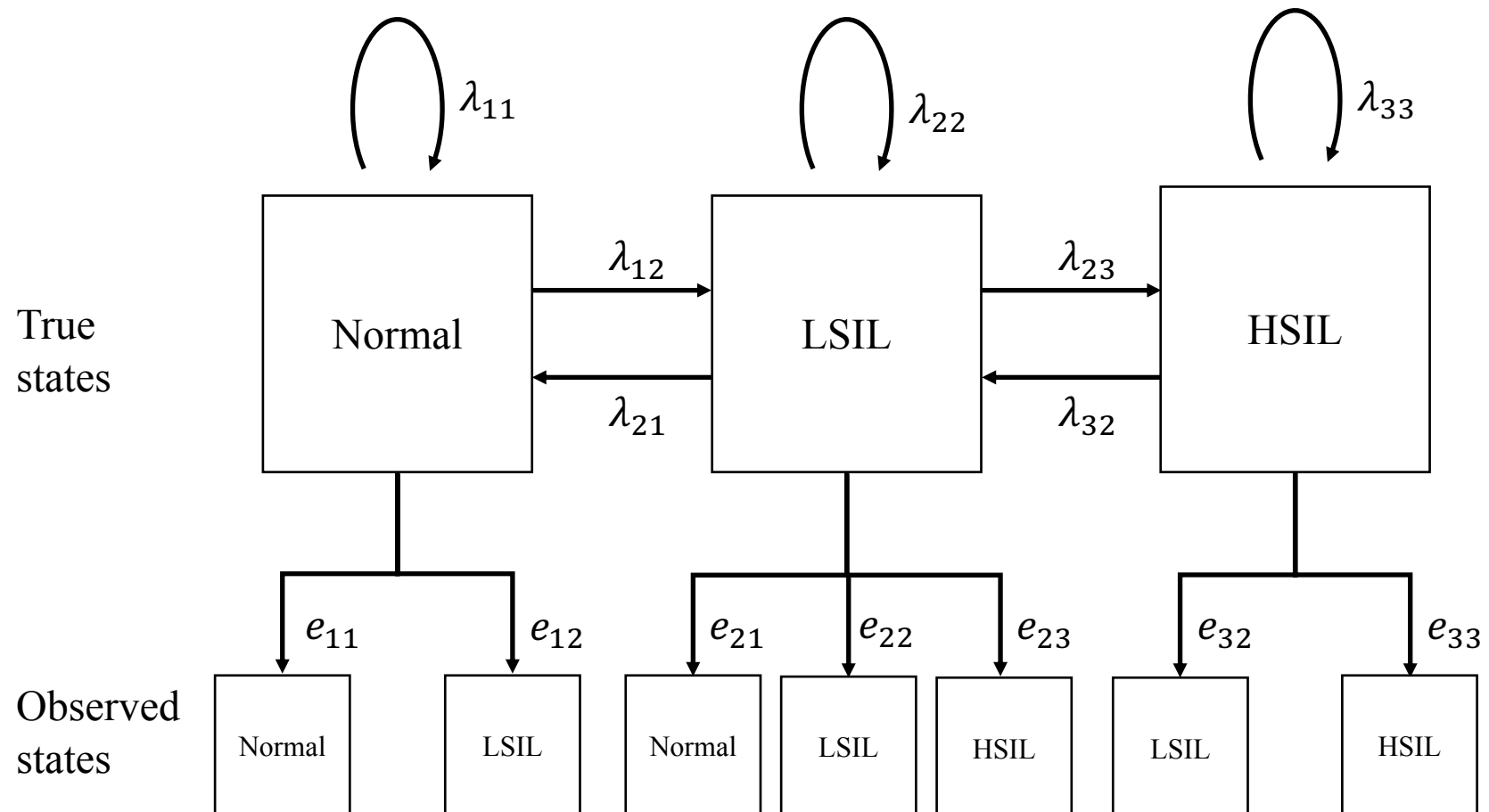

Supplement: Supplementary file 2 — Fig S2 [file CAM4-11-664-s003.pdf]

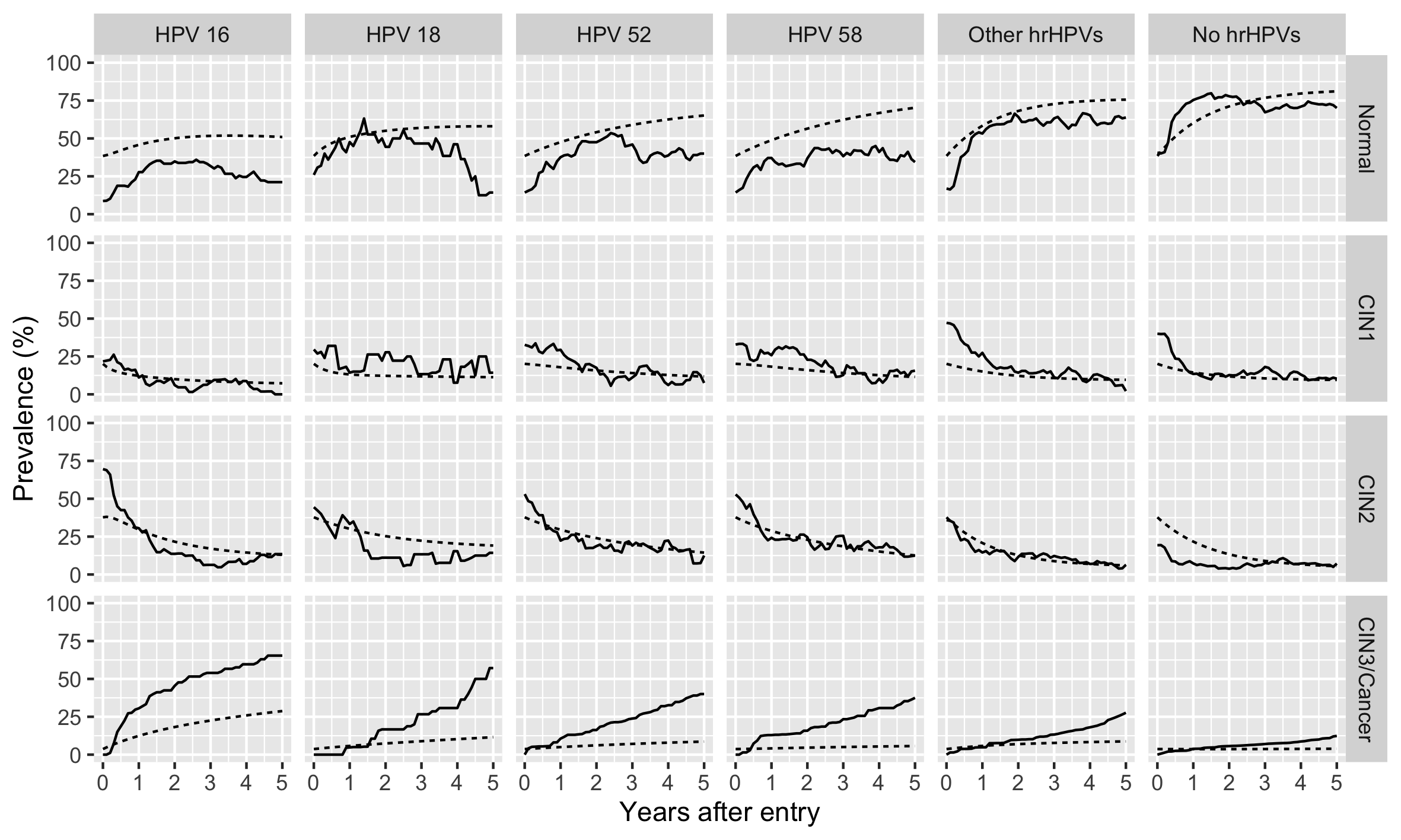

Supplement: Supplementary file 3 — Fig S3 [file CAM4-11-664-s002.tiff]

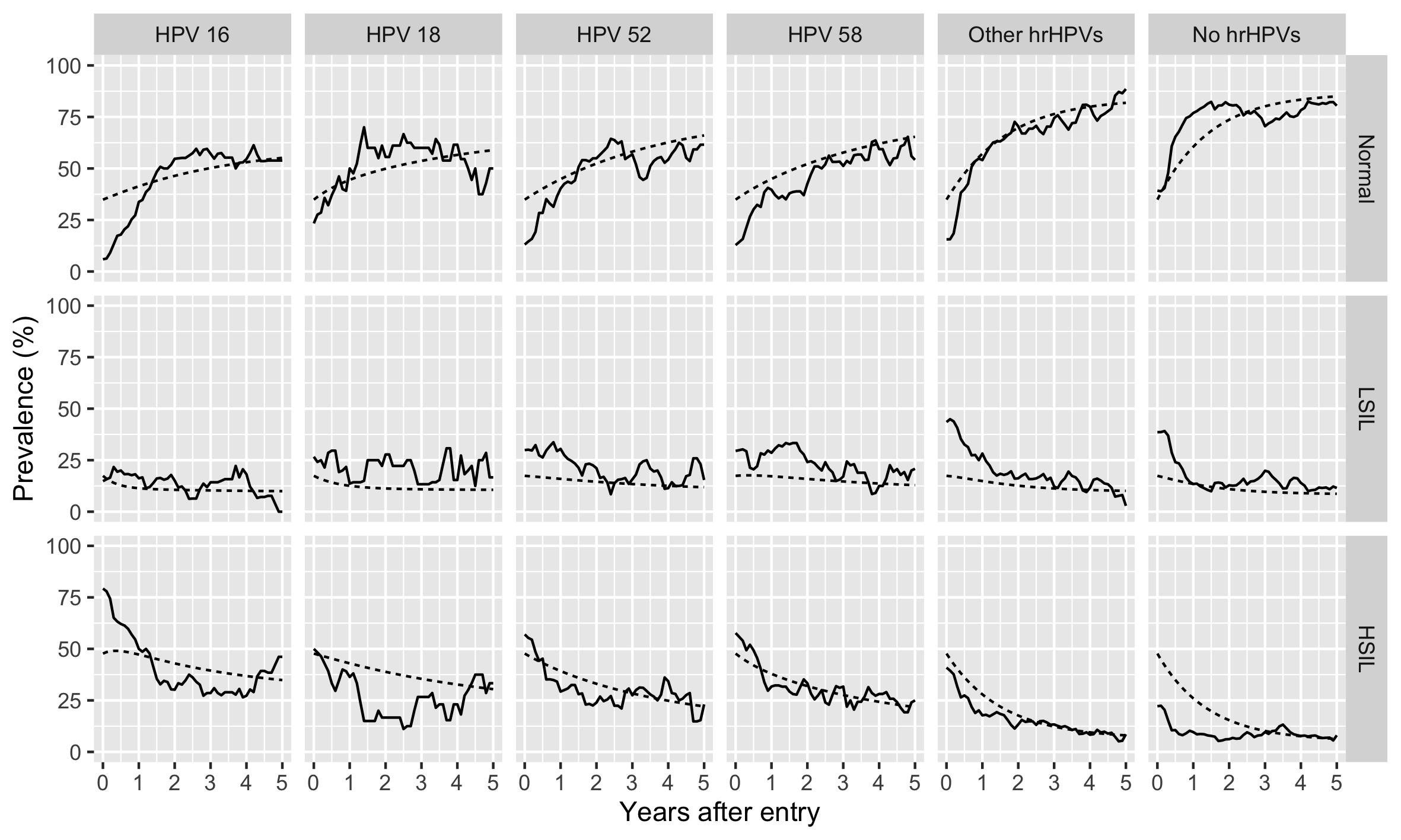

Supplement: Supplementary file 4 — Fig S4 [file CAM4-11-664-s004.tiff]
