# Supplementary material for: Prognosis of high‐risk human papillomavirus‐related cervical lesions: A hidden Markov model analysis of a single‐center cohort in Japan
Source: Cancer Med. 2021 Dec 17;11(3):664–75. doi: 10.1002/cam4.4470 (PMC8817087; doi:10.1002/cam4.4470)
Supplement: Supplementary file 5 — Table S1‐S6 [file CAM4-11-664-s001.doc]

**Supplementary tables**

Table S1. Misclassification probabilities for CIN stages based on cytology and histology.

| True underlying state | Observed state | | | |
| --- | --- | --- | --- | --- |
|  | Normal | CIN1 | CIN2 | CIN3/cancer |
| Normal | 0.971 (0.954–0.982) | 0.028 (0.017–0.045) | 0.000 (0.000–0.000) | 0.000 (0.000–0.000) |
| CIN1 | 0.268 (0.203–0.343) | 0.642 (0.525–0.744) | 0.089 (0.055–0.139) | 0.000 (0.000–0.000) |
| CIN2 | 0.000 (0.000–0.000) | 0.074 (0.048–0.111) | 0.925 (0.888–0.951) | 0.000 (0.000–0.000) |
| CIN3/cancer | 0.000 (0.000–0.000) | 0.000 (0.000–0.000) | 0.114 (0.033–0.323) | 0.885 (0.676–0.966) |

Values are the estimated emission probabilities (95% confidence interval).

Table S2. Predicted two-year transition probabilities according to HPV genotype.

| Current state | HPV category | State after two years | | | |
| --- | --- | --- | --- | --- | --- |
|  |  | Normal | CIN1 | CIN2 | CIN3/cancer |
| Normal | HPV 16 | 0.800 (0.667–0.894) | 0.072 (0.037–0.128) | 0.082 (0.040–0.142) | 0.044 (0.021–0.082) |
|  | HPV 18 | 0.713 (0.476–0.880) | 0.190 (0.074–0.343) | 0.077 (0.020–0.184) | 0.018 (0.003–0.064) |
|  | HPV 52 | 0.808 (0.663–0.902) | 0.120 (0.060–0.208) | 0.059 (0.028–0.114) | 0.012 (0.005–0.027) |
|  | HPV 58 | 0.937 (0.729–0.986) | 0.041 (0.008–0.179) | 0.017 (0.003–0.084) | 0.002 (0.000–0.017) |
|  | Other hrHPVs | 0.859 (0.775–0.918) | 0.114 (0.066–0.181) | 0.020 (0.010–0.040) | 0.005 (0.002–0.011) |
|  | No hrHPVs | 0.865 (0.824–0.901) | 0.098 (0.072–0.130) | 0.029 (0.019–0.042) | 0.005 (0.003–0.008) |
| CIN1 | HPV 16 | 0.410 (0.303–0.525) | 0.107 (0.068–0.162) | 0.205 (0.142–0.268) | 0.277 (0.192–0.375) |
|  | HPV 18 | 0.620 (0.398–0.753) | 0.189 (0.104–0.349) | 0.132 (0.049–0.278) | 0.056 (0.017–0.164) |
|  | HPV 52 | 0.438 (0.338–0.532) | 0.253 (0.192–0.329) | 0.215 (0.156–0.284) | 0.092 (0.056–0.146) |
|  | HPV 58 | 0.375 (0.271–0.497) | 0.318 (0.230–0.421) | 0.224 (0.154–0.301) | 0.081 (0.043–0.136) |
|  | Other hrHPVs | 0.727 (0.647–0.786) | 0.179 (0.135–0.235) | 0.059 (0.034–0.094) | 0.034 (0.017–0.062) |
|  | No hrHPVs | 0.667 (0.624–0.705) | 0.183 (0.157–0.213) | 0.104 (0.085–0.127) | 0.045 (0.032–0.063) |
| CIN2 | HPV 16 | 0.239 (0.161–0.336) | 0.104 (0.066–0.151) | 0.217 (0.149–0.293) | 0.437 (0.336–0.550) |
|  | HPV 18 | 0.260 (0.099–0.464) | 0.137 (0.057–0.266) | 0.320 (0.117–0.535) | 0.281 (0.113–0.557) |
|  | HPV 52 | 0.258 (0.183–0.354) | 0.259 (0.196–0.325) | 0.267 (0.195–0.346) | 0.214 (0.134–0.311) |
|  | HPV 58 | 0.233 (0.152–0.326) | 0.331 (0.249–0.413) | 0.259 (0.180–0.350) | 0.175 (0.104–0.293) |
|  | Other hrHPVs | 0.452 (0.347–0.544) | 0.206 (0.157–0.261) | 0.135 (0.079–0.211) | 0.205 (0.120–0.324) |
|  | No hrHPVs | 0.415 (0.361–0.467) | 0.218 (0.187–0.247) | 0.188 (0.149–0.232) | 0.177 (0.136–0.236) |

hrHPV: high-risk human papillomavirus.

Values are the predicted transition probabilities (95% confidence interval) from the current true lesions over two years.

Other hrHPVs included HPV 31, 33, 35, 39, 45, 51, 56, 59, and 68.

No hrHPVs were HPVs other than HPV 16, 18, 52, 58, or other hrHPVs.

Table S3. Basic characteristics of the study subjects at the time of entry (two-tier classification).

| Diagnosis at the time of entry |  | HPV 16 | HPV 18 | HPV 52 | HPV 58 | Other hrHPVs | No hrHPVs | All |
| --- | --- | --- | --- | --- | --- | --- | --- | --- |
| Normal | N | 8 | 7 | 14 | 10 | 24 | 122 | 185 |
|  | Age at entry (years), mean (SD) | 39.9 (8.2) | 45.3 (15.9) | 38.0 (10.1) | 44.9 (17.4) | 42.7 (16.3) | 41.2 (10.5) | 41.5 (11.9) |
|  | Number of visits, mean (SD) | 9.1 (6.9) | 8.5 (2.5) | 8.9 (7.2) | 7.0 (5.0) | 8.0 (4.8) | 6.6 (3.9) | 7.1 (4.5) |
|  | Follow-up interval (years), mean (SD) | 0.47 (0.33) | 0.51 (0.31) | 0.50 (0.36) | 0.46 (0.41) | 0.46 (0.26) | 0.52 (0.39) | 0.50 (0.36) |
|  | Follow-up period (years), mean (SD) | 3.6 (2.9) | 3.5 (1.0) | 4.3 (3.3) | 3.0 (2.4) | 3.4 (2.2) | 3.1 (2.1) | 3.3 (2.2) |
| LSIL | N | 20 | 8 | 32 | 23 | 67 | 120 | 270 |
|  | Age at entry (years), mean (SD) | 35.3 (9.2) | 33.0 (10.6) | 39.1 (8.7) | 36.7 (8.3) | 34.5 (7.1) | 38.8 (10.1) | 37.1 (9.2) |
|  | Number of visits, mean (SD) | 9.6 (5.3) | 6.5 (2.8) | 10.3 (6.0) | 11.1 (5.4) | 9.5 (4.5) | 9.4 (5.3) | 9.6 (5.2) |
|  | Follow-up interval (years), mean (SD) | 0.38 (0.17) | 0.49 (0.54) | 0.39 (0.21) | 0.41 (0.28) | 0.38 (0.18) | 0.42 (0.30) | 0.40 (0.26) |
|  | Follow-up period (years), mean (SD) | 3.7 (2.5) | 3.0 (1.8) | 3.9 (2.3) | 4.2 (2.4) | 3.6 (2.0) | 3.9 (2.4) | 3.8 (2.3) |
| HSIL | N | 107 | 15 | 61 | 45 | 63 | 69 | 360 |
|  | Age at entry (years), mean (SD) | 37.2 (8.0) | 42.5 (5.6) | 40.9 (7.8) | 40.2 (7.9) | 39.6 (7.9) | 37.2 (8.9) | 38.8 (8.2) |
|  | Number of visits, mean (SD) | 6.7 (6.1) | 9.7 (8.0) | 8.4 (6.3) | 9.5 (5.8) | 8.3 (5.6) | 9.4 (5.7) | 8.2 (6.1) |
|  | Follow-up interval (years), mean (SD) | 0.31 (0.25) | 0.31 (0.12) | 0.34 (0.18) | 0.35 (0.22) | 0.36 (0.38) | 0.34 (0.32) | 0.33 (0.27) |
|  | Follow-up period (years), mean (SD) | 1.9 (2.2) | 3.2 (3.3) | 2.8 (2.5) | 3.4 (2.4) | 2.9 (2.3) | 3.4 (2.3) | 2.8 (2.4) |

hrHPV: high-risk human papillomavirus. SD: standard deviation.

Other hrHPVs included HPV 31, 33, 35, 39, 45, 51, 56, 59, and 68. No hrHPVs were HPVs other than HPV 16, 18, 52, 58, or other hrHPVs.

Table S4. Transitions from each diagnosis of cervical epithelial lesions according to HPV genotype (two-tier classification)

| Diagnosis at (t-1) visit | HPV category | Diagnosis at t visit | | | |
| --- | --- | --- | --- | --- | --- |
|  |  | Normal | LSIL | HSIL | Cancer |
| Normal | HPV 16 | 236 (86.7) | 13 (4.7) | 23 (8.4) | 0 (0.0) |
|  | HPV 18 | 81 (84.3) | 11 (11.4) | 4 (4.1) | 0 (0.0) |
|  | HPV 52 | 259 (78.0) | 44 (13.2) | 29 (8.7) | 0 (0.0) |
|  | HPV 58 | 230 (83.6) | 24 (8.7) | 21 (7.6) | 0 (0.0) |
|  | Other hrHPVs | 598 (86.9) | 72 (10.4) | 18 (2.6) | 0 (0.0) |
|  | No hrHPVs | 1383 (89.9) | 122 (7.9) | 32 (2.0) | 1 (0.0) |
| LSIL | HPV 16 | 26 (22.2) | 39 (33.3) | 52 (44.4) | 0 (0.0) |
|  | HPV 18 | 16 (40.0) | 14 (35.0) | 10 (25.0) | 0 (0.0) |
|  | HPV 52 | 65 (31.7) | 89 (43.4) | 51 (24.8) | 0 (0.0) |
|  | HPV 58 | 43 (27.2) | 75 (47.4) | 40 (25.3) | 0 (0.0) |
|  | Other hrHPVs | 126 (46.1) | 113 (41.3) | 34 (12.4) | 0 (0.0) |
|  | No hrHPVs | 228 (52.4) | 164 (37.7) | 43 (9.8) | 0 (0.0) |
| HSIL | HPV 16 | 35 (7.5) | 52 (11.2) | 373 (80.3) | 4 (0.8) |
|  | HPV 18 | 8 (8.6) | 9 (9.7) | 75 (81.5) | 0 (0.0) |
|  | HPV 52 | 39 (11.9) | 47 (14.4) | 240 (73.6) | 0 (0.0) |
|  | HPV 58 | 28 (11.3) | 46 (18.6) | 171 (69.5) | 1 (0.4) |
|  | Other hrHPVs | 45 (18.9) | 38 (15.9) | 154 (64.7) | 1 (0.4) |
|  | No hrHPVs | 62 (20.3) | 46 (15.0) | 197 (64.5) | 0 (0.0) |

hrHPV: high-risk human papillomavirus.

Values are the number (percentage) of observed transitions from a visit to the next visit.

Other hrHPVs included HPV 31, 33, 35, 39, 45, 51, 56, 59, and 68.

No hrHPVs were HPVs other than HPV 16, 18, 52, 58, or other hrHPVs.

Table S5. Misclassification probabilities for the two-tier classification based on cytology and histology.

| True underlying state | Observed state | | |
| --- | --- | --- | --- |
|  | Normal | LSIL | HSIL |
| Normal | 0.966 (0.951–0.977) | 0.033 (0.022–0.048) | 0.000 (0.000–0.000) |
| LSIL | 0.247 (0.193–0.310) | 0.616 (0.528–0.698) | 0.135 (0.098–0.184) |
| HSIL | 0.000 (0.000–0.000) | 0.045 (0.028–0.069) | 0.954 (0.930–0.971) |

Values are the estimated emission probabilities (95% confidence interval).

Table S6. Predicted two-year transition probabilities according to HPV genotype (two-tier classification).

| Current state | HPV category | State after two years | | |
| --- | --- | --- | --- | --- |
|  |  | Normal | LSIL | HSIL |
| Normal | HPV 16 | 0.810 (0.678–0.892) | 0.082 (0.044–0.140) | 0.107 (0.057–0.196) |
|  | HPV 18 | 0.829 (0.542–0.956) | 0.124 (0.033–0.310) | 0.046 (0.007–0.178) |
|  | HPV 52 | 0.899 (0.672–0.974) | 0.067 (0.018–0.208) | 0.033 (0.008–0.114) |
|  | HPV 58 | 0.903 (0.754–0.967) | 0.066 (0.022–0.171) | 0.029 (0.009–0.079) |
|  | Other hrHPVs | 0.867 (0.787–0.918) | 0.111 (0.067–0.178) | 0.020 (0.009–0.041) |
|  | No hrHPVs | 0.878 (0.834–0.912) | 0.089 (0.065–0.122) | 0.031 (0.021–0.045) |
| LSIL | HPV 16 | 0.448 (0.338–0.555) | 0.135 (0.097–0.196) | 0.416 (0.313–0.520) |
|  | HPV 18 | 0.605 (0.332–0.773) | 0.190 (0.084–0.426) | 0.203 (0.073–0.407) |
|  | HPV 52 | 0.420 (0.313–0.518) | 0.288 (0.224–0.372) | 0.291 (0.219–0.371) |
|  | HPV 58 | 0.372 (0.280–0.489) | 0.341 (0.262–0.433) | 0.285 (0.206–0.378) |
|  | Other hrHPVs | 0.732 (0.652–0.788) | 0.191 (0.147–0.259) | 0.075 (0.043–0.123) |
|  | No hrHPVs | 0.646 (0.604–0.682) | 0.199 (0.171–0.229) | 0.154 (0.127–0.181) |
| HSIL | HPV 16 | 0.224 (0.151–0.305) | 0.158 (0.113–0.217) | 0.617 (0.515–0.708) |
|  | HPV 18 | 0.160 (0.047–0.337) | 0.144 (0.053–0.306) | 0.695 (0.422–0.884) |
|  | HPV 52 | 0.232 (0.159–0.318) | 0.326 (0.260–0.397) | 0.441 (0.350–0.540) |
|  | HPV 58 | 0.222 (0.153–0.307) | 0.379 (0.303–0.456) | 0.397 (0.304–0.499) |
|  | Other hrHPVs | 0.479 (0.361–0.576) | 0.268 (0.213–0.333) | 0.251 (0.157–0.386) |
|  | No hrHPVs | 0.393 (0.343–0.446) | 0.267 (0.238–0.298) | 0.338 (0.287–0.395) |

hrHPV: high-risk human papillomavirus.

Values are the predicted transition probabilities (95% confidence interval) from the current true lesions over two years.

Other hrHPVs included HPV 31, 33, 35, 39, 45, 51, 56, 59, and 68.

No hrHPVs were HPVs other than HPV 16, 18, 52, 58, or other hrHPVs.
