# Supplementary material for: Prognosis of high‐risk human papillomavirus‐related cervical lesions: A hidden Markov model analysis of a single‐center cohort in Japan
Source: Cancer Med. 2021 Dec 17;11(3):664–75. doi: 10.1002/cam4.4470 (PMC8817087; doi:10.1002/cam4.4470)
Supplement: Supplementary file 6 — Appendix [file CAM4-11-664-s006.doc]

**eAppendix 1.**

For the sensitivity analysis according to the two-tier classification, we constructed a different dataset that included patients (1) diagnosed with a normal cervical lesion, LSIL, or HSIL at the time of entry and (2) observed for at least two visits during the follow-up period. Patients were excluded if they had HPV 6-single-positive lesions with the sole diagnosis of condyloma during their follow-up period and those with only glandular lesions. Patients with multiple HPV genotypes were excluded. One patient with malignant lymphoma was also excluded. Patients were followed-up until they received treatment, were diagnosed with cervical cancer, were moved to another hospital, or until March 31, 2018, whichever occurred first. Finally, 815 patients (6915 observations) were included in the two-tier classification (Figure S1).

In this dataset, we translated the CIN categories into the two-tier classification by mapping normal to normal, CIN1 to LSIL, and CIN2 and CIN3/cancer to HSIL/cancer. Other variables were defined in the same manner as in the primary analysis.

**eAppendix 2.**

For the two-tier classification, at the time of entry, 185 (22.7%), 270 (33.1%), and 360 (44.2%) patients were in the normal, LSIL, and HSIL categories, respectively (Table S1). The progression from LSIL to HSIL was dependent on the HPV genotype; 44.4%, 25.0%, 24.8%, 25.3%, 12.4%, and 9.8% with HPV 16, 18, 52, and 58 genotypes, other hrHPVs, and no hrHPVs, respectively, progressed to HSIL. Overall, HPV 16-positive patients were more likely to progress to more severe states than patients with other HPV genotypes (Table S2).
